# Assessment of the nevirapine safety signal using data from the national antiretroviral dispensing database: a retrospective study

**DOI:** 10.1186/s40545-016-0054-x

**Published:** 2016-02-15

**Authors:** Francis Kalemeera, Assegid T. Mengistu, Johannes Gaeseb

**Affiliations:** School of Pharmacy, Faculty of Health Sciences, University of Namibia, Windhoek, Namibiaᅟ; Therapeutics Information and Pharmacovigilance Centre, Namibia Medicines Regulatory Council, Ministry of Health and Social Services, Windhoekᅟ, Namibia; Registrar of Medicines, Namibia Medicines Regulatory Council, Ministry of Health and Social Services, Windhoek, Namibia

**Keywords:** Nevirapine, Reaction ratio, Proportional reaction ratio, Adverse reaction, Signal, Skin, Liver, Grade III, Grade IV

## Abstract

**Background:**

Clinical trials showed a higher risk of skin- and liver- related adverse reactions when NVP-based antiretroviral therapy (ART) was initiated in female and male patients with baseline CD4 cell counts ≥250 and ≥400, respectively. Some studies reported no difference in risk between the high and low CD4 count groups. Consequently, the use of NVP-based ART in all patients with a CD4 cell count <350, was recommended. In 2011, the Pharmacovigilance Centre detected an increase in reports of grade III and IV reactions. The center was required to determine if there was an increase in NVP-related reactions.

**Methods:**

Automated dispensing records from January 2008 to November 2011 were accessed from the National Antiretroviral Dispensing Database (NDB). Records of patients who were initiated on NVP-based ART were selected, and records showing a replacement of NVP with protease inhibitor (PI) were identified. The proportions of grade III and IV reactions were calculated per quarter, and Odds Ratios (OR) were calculated, with the confidence interval set at 95 % and a p-value of <0.05.

**Results:**

From 2008 to 2011 a total of 84,741 patients were started on ART. Of these 67,794 were initiated on NVP-containing ART. Of these, 211 females and 79 males were substituted from NVP to a PI. The OR for females was 2.4 (95 % confidence interval [CI] 1.8 – 3.1). For males the OR was 2.4 (OR 2.4; 95 % CI 1.4 – 3.8) which occurred nine months after the change observed in the females. The odds of a NVP-to-PI substitution in females compared to males before the launch of Namibia’s 2010 ART guidelines was the same as the odds after the publication of the guidelines (before, OR 1.6; 95 % CI 1.1 – 2.5; after, OR 1.6; 95 % CI 1.2 – 2.2).

**Conclusions:**

There was an increase in substitutions of NVP with a PI following the increase in the CD4 threshold for initiating NVP-based HAART, meaning that there was an increase in grade III and IV reactions associated with NVP. Therefore the NVP-safety signal was confirmed to be a true signal, which contributed to the Ministry’s decision to review the use of NVP.

## Background

### Guidelines on managing NVP-related adverse reactions

Nevirapine (NVP) is a non-nucleoside reverse transcriptase inhibitor (NNRTI) used in combination with other antiretroviral medicines for the treatment of people infected with the human immunodeficiency virus (HIV) [1]. The use of NVP-containing antiretroviral therapy (ART) has proven its worth as evidenced by the significant reductions in HIV-associated morbidity and mortality in regions where NVP-containing ART regimens were preferred [2]. Nevertheless, NVP is known to cause adverse reactions (AR) on the skin and in the liver, which necessitate NVP’s substitution if the reaction is severe or persists without resolution. Namibia’s ART guidelines have consistently recommended the continuation of NVP, with observation, for the patient who experienced a grade I AR to NVP; the substitution of NVP with efavirenz for the patient who experienced a grade II AR; and the substitution of NVP with lopinavir/ritonavir (LPV/r) a protease inhibitor, for the patient who experienced grade III or IV ARs to NVP [3] (See Table 1).

**Table 1:** Namibia ART guidelines 2008, 2010, and 2014 on the Use of NVP and response to NVP-related adverse reactions

Year of Guidelines	CD4 count threshold for ART initiation	Preferred ART regimen	Response following NVP-related adverse drug reaction (skin and liver)	Recommended Second Line ART
Pre-2008	<200cells/mm^3^	D4T/3TC/NVP	• Grade I observe, add some medicine	TDF/3TC/LPV/r
2008	<200cells/mm^3^	AZT/3TC/NVP		TDF/3TC/LPV/r
2010	≤350cells/mm^3^	TDF/3TC/NVP	• Grade II, replace NVP with EFV	TDF/3TC/AZT/LPV/r
			• Grade III or IV, replace NVP with LPV/r	
2014	≤500cells/mm^3^	TDF/FTC/EFV	Left blank – focus is placed on NVP	AZT/FTC/LPV/r

*D4T* Stavudine, *3TC* Lamivudine, *NVP* Nevirapine, *AZT* Zidovudine, *TDF* Tenofovir, *FTC* Emtricitabine, *EFV* Efavirenz, *LPV/r* Lopinavir/ritonavir

### NVP-use recommended in ART naïve patients with high baseline CD4 cell counts

NVP’s prescribing information has for long recommended the avoidance of NVP in women and men with CD4 cell counts ≥ 250 and ≥400 cells/mm^3^ respectively. This safety measure was devised to reduce incidences of serious NVP-related AR on the skin and in the liver [4]. Over the years, this safety measure was countered by a number of studies that have suggested that the incidence of NVP-related skin and liver AR in patients with high baseline CD4 cell counts – ≥250 in females and ≥400 in males – was not different from the incidence in patients with low baseline CD4 counts [5–12]. On the contrary, results from other similar studies maintained that the incidence of NVP-related skin and liver reactions was higher in patients starting NVP-based antiretroviral therapy (ART) at high baseline CD4 cell counts than in patients with low baseline CD4 counts [13–17]. Nevertheless, based on the then available evidence, the World Health Organisation (WHO) recommended the use of NVP-based ART in all patients with a CD4 cell count <350 cells/mm^3^, including ART naïve females with a CD4 count >250 but <350cells/mm^3^ [1]. In 2010, Namibia’s Ministry of Health and Social Services (MoHSS) launched the Third Edition of the National Guidelines for ART, which emulated the WHO’s ART guidelines in regards to the threshold CD4 count for initiating ART, and the preferred first line ART regimen, namely: tenofovir (TDF)/lamivudine (3TC) and NVP [3]. Also, the guidelines recommended close monitoring of liver function and careful examination of skin and mucosal surfaces of patients during the first weeks of NVP-containing ART, especially for females with baseline CD4 counts greater than 250 cells/mm^3^ [1, 3]. (See Table 1).

### Detection of a significant increase in spontaneous reports of grade III and IV AR related to NVP: Signal detection

About one year after the launch of Namibia’s 2010 ART Guidelines the Therapeutics Information and Pharmacovigilance Centre (TIPC) carried out an analysis of the spontaneous AR reports. They compared the numbers and proportions of reports that were received from 2008 to 2010 – that is before the 2010 ART guidelines – with those that were received in 2011. The results showed a statistically significant increase in the reports of grade III and IV skin and liver reactions associated with NVP in 2011 compared with previous years (2008 to 2010) (*p* < 0.0001). In contrast to this result, there was no change in the frequency of grade I and II reactions (*P* = 0.16) between the periods. TIPC concluded that the increase in frequency of NVP-related grade III and IV reactions was a signal of the increase in grade III and IV ARs associated with NVP [18]. (See Appendix 1: Table 5 for the results obtained during the signal detection phase of NVP-safety analysis, and Appendix 2: Table 6 for the statistical significance of the results). The skin- and liver- related reactions reports for NVP were compared with EFV-related reaction reports. This comparison showed that there was no change in the EFV-related reports, proving that the increase in reaction reports was unique to NVP. Similarly, the number of reports for EFV and NVP were compared with the number of reports for other medicines. This comparison showed that the increase in reports in 2011 was a finding unique to NVP. The within medicine comparison (NVP before and NVP after the 2010 ART guidelines) and the between medicine comparisons were done using Reaction Ratio, and Proportional Reaction Ratio [18]. (See Appendix 3: Tables 7 and 8 for the comparisons between NVP related reaction reports before and after the 2010 ART guidelines, and for the comparison between other medicines and EFV, and NVP).

### The need to confirm or dispel the NVP-safety signal: TIPC’s mandate

Since the above findings were based on spontaneous reports that were sent to the pharmacovigilance center voluntarily no conclusions could be drawn about the frequency of grade III and IV reactions at clinic level. (See the legend of Appendix 1: Table 5 for the actual reactions that were reported to TIPC.) The clinical importance [19] of these reactions required the TIPC to carry out further analysis to generate evidence that would confirm or dispel the NVP safety signal.

In this paper we present the objectives methods, and results that were generated from the research activity that TIPC undertook to assess the NVP-safety signal. Also, we present the discussion of the findings, and highlight the associated policy changes that followed. We believe that this paper demonstrates that local pharmacovigilance activities, based on locally generated data are beneficial to local populations.

## Objective

The objective of this assessment was to find out if the increase in spontaneous reports of grade III and IV reactions associated with NVP was a true reflection of an increase in grade III and IV reaction events at clinic level. We set out to estimate the incidence of substitution of NVP with a protease inhibitor (PI) during the period 2008 to 2011 and to determine if any increase detected was statistically significant.

## Methods

Automated antiretroviral (ARV) medicine dispensing records for adult patients who were newly started on ART during the period January 1 2008 to November 30, 2011 were accessed from the National Antiretroviral Dispensing Database. The proportion of records that showed the substitution of NVP with a PI per quarter from January 2008 to November 2011 was calculated. Comparisons between and within gender were made using the Odds Ratio, Proportional Reporting Ratio, and Reporting Ratio, with the confidence interval and statistical significance set at 95 % and a p-value of <0.05, respectively. (In this paper the substitution of NVP with a PI is frequently referred to as a NVP-to-PI substitution. The PI that was mainly used is LPV/r).

### Ethical approval

Patient consent was not sought because antiretroviral data was sourced from existing databases and clinical records without requiring additional information directly from patients. ART dispensing data was captured in the national database housed in the Ministry of Health and Social Services’ Division of Pharmaceutical Services. Only TIPC staff members mandated by the MoHSS to implement medicines safety analyses were involved in the medical record abstraction process and in the epidemiological study considered to be a public health activity. Confidentiality was observed.

## Results

### Frequency

**Fig. 1:**
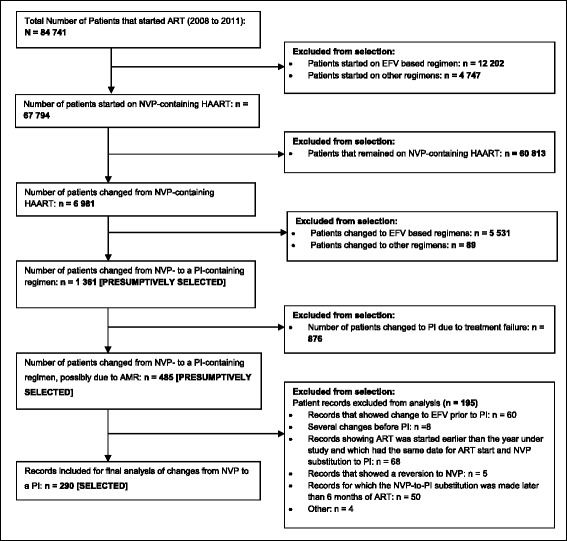
Selection of records with a NVP-to-PI substitution

A total of 84 741 patients were initiated on ART from January 2008 to November 2011. Of these, 67 794 were initiated on a NVP-containing regimen. For 290 patients NVP was substituted with a PI (Fig. 1). The period from the start of ART to when NVP was replaced with a PI, and the number of patients in whom this happened is shown in Table 2. The mean (and median) ages of the males and females were 41 (39) years and 36 (35) years, respectively.

**Table 2:** Number of patients with a NVP-to-PI substitution

Time after starting ART (number of weeks)	Number of NVP to PI substitutions				Total
	2008	2009	2010	2011	
2	3	7	2	12	24
4	1	6	23	12	42
6	6	8	24	32	70
8	4	6	11	26	47
10	1	4	7	12	24
12	2	4	9	10	25
14	3	5	6	3	17
16	3	2	3	4	12
18	0	1	2	4	7
20	1	4	3	4	12
22	1	1	1	2	5
24	0	0	2	3	5
Total	25	48	93	124	290

Throughout the period that was assessed, the majority of NVP-to-PI substitutions took place within the first12 weeks of ART. Higher numbers of substitutions are observed in 2010 and even higher in 2011 than the two previous years

### The Frequency of ART initiation

**Fig. 2:**
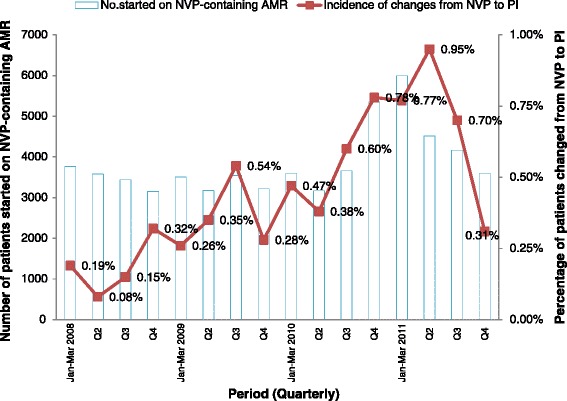
The figure shows that in the last quarter of 2010, the number of NVP-to-PI substitutions exceeded those in the previous quarters, as did the number of patients newly started on ART (*n* = 5 352). These substitutions peaked in the first quarter of 2011, but dropped in the subsequent two quarters. In the last quarter of 2011, the number of patients started on ART reverted to the earlier total level (*n* = 3 025)

From the first quarter of 2008 to the third quarter of 2010 the average number of HIV- positive patients who were initiated on ART per quarter was 3 437 (Fig. 2). There was an increase in this number in the third quarter of 2010.

### Proportion of patients with a NVP- to-PI substitutions

From the second quarter of 2010, the frequency of NVP-to-PI substitutions rose constantly to a peak of 0.95 % in the second quarter of 2011. In the third quarter of 2011 the frequency dropped slightly to 0.70 %, and then sharply by the last quarter of 2011 to 0.20 % (Fig. 2).

### Increase in the Use of TDF-based first line HAART

**Fig. 3:**
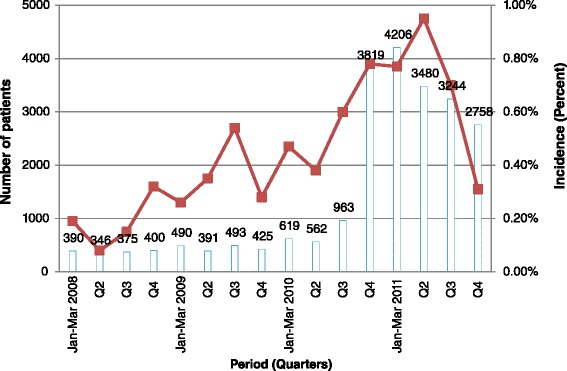
A major increase in TDF as a part of the nucleoside backbone in first line antiretroviral therapy is observed in the last quarter of 2010. Although a reduction is observed in the consequent months, the use of TDF remained high compared to what it was in the earlier quarters

An increase in the use of TDF-based HAART is observed in the last two quarters of 2010 (Fig. 3).

### Trends in the NVP-to-PI Substitutions according to gender

The majority of NVP-to-PI substitutions occurred in females (72.7 %, *n* = 211).

### Females

The proportion of NVP-to-PI substitutions peaked in the third quarter of 2010. The odds of getting a NVP-to-PI substitution in the third quarter of 2010 and afterwards was 2.4 times the odds of getting the same substitution in the earlier period (January 2008 to June 2010) (95 % CI 1.8–3.1; *p* < 0.0001) (Table 3).

**Table 3:** Comparison of Frequencies of NVP-to-PI Substitutions before and after July 2010: Results According to Gender

	No. Started ART before Q3 2010	No. changed from NVP to PI	No. Started ART in and after Q3 2010	No. changed from NVP to PI	Odds Ratio (CI)	*P*-value
Females	21 034	74	16 540	137	2.4 (1.8–3.1)	<0.0001
Males	13 012	28	9 987	51	2.4 (1.4–3.8)	0.0002

### Males

**Fig. 4:**
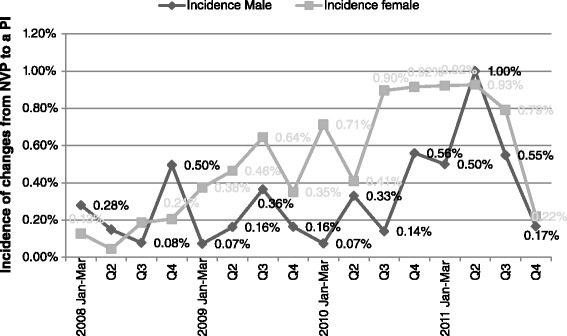
Shows an increase in NVP-to-PI substitution in the third quarter of 2010, which was maintained for the consequent three quarters. We observed a decline in NVP-to-PI substitutions in the third quarter of 2011

The NVP-to-PI substitutions in males fluctuated through the entire period with a spike in the second quarter of 2011 (Fig. 4). The odds of needing a NVP-to-PI substitution from the third quarter of 2010 and afterwards was 2.4 times the odds of getting the same substitution in the earlier period (January 2008 to June 2010) (95 % CI 1.4–3.8; *p* = 0.0002) (Table 3).

Before and after July 2010, the odds of needing a NVP-to-PI substitution in females was 1.6 times that for males (*P* <0.05) (Table 4).

**Table 4:** Number of NVP-to-PI Substitutions for females vs. males before and after the Launch of the 2010 ART Guidelines

Period	Males		Females		Odds Ratio (CI)	*P*-value
	No. started on ART	No changed from NVP to PI	No. started on ART	No. changed from NVP to PI		
July 2010 and after	9,987	51	16,540	137	1.6 (1.2–2 .2)	0.003
Before July 2010	13,012	28	21,034	74	1.6 (1.1–2.5)	0.03

## Discussion

The assumption that the substitution of NVP with a PI (mainly LPV/r) within 24 weeks of ART was made due to grade III or IV ARs, was substantiated by the following. First: NVP-related reactions of liver and skin are known to occur mainly within the first few weeks of ART, but they may also occur as late as 18 weeks and more [4, 16]. Second: Namibia’s 2010 ART guidelines recommended that when a grade III or IV AR – such as Steven-Johnson’s Syndrome, Toxic Epidermal Necrolysis, and very high alanine transferase levels in plasma (hepatotoxicity) – occurs, NVP should be substituted with LPV/r [3]. Third: The replacement of NVP with LPV/r, without any alteration in the nucleoside backbone, within six months of ART was not judged to be treatment failure. Namibia’s 2008 ART guidelines recommended adherence-counselling for patients with poor virologic response before, at, or after six months of ART [3]. Therefore, TDF/3TC/LPV/r was considered to be a first line regimen.

During the period before the publication of Namibia’s 2010 ART guidelines, TDF had been prescribed as part of the nucleoside backbone of first line ART only when zidovudine or stavudine were contraindicated [20]. Immediately after the publication of these guidelines, there was a substantial increase in the dispensing of TDF/3TC/NVP, the new preferred first line regimen. This immediate increase in the dispensing of TDF/3TC/NVP was not surprising, because the coordination between the Division of Pharmaceutical Services (DPS) and the Directorate of Special Programs (DSP) allowed the publication of the guidelines only when the medicines were available at the Central Medical Stores. [The DPS is in charge of the supply chain management of all pharmaceuticals for the Ministry of Health; and DSP is responsible for HIV care and treatment.] Therefore, compliance to the ART guidelines on issues such as the choice of first line ART regimen was facilitated, on the big part, by availability of the regimen, but also by the training of health care workers (HCW), who work in health facilities that provide ART.

The publication of the 2010 ART guidelines was followed by the increase in the number of patients who were newly initiated on ART. The previous guidelines recommended that one was eligible for ART when one’s CD4 count was <200cells/mm^3^. During that period (2008–Early 2010), an average of 3,414 patients were initiated on first line ART per quarter (Range = 613). The 2010 ART guidelines recommended that one was eligible for ART when one’s CD4 count was ≤350cells/mm^3^. After the 2010 ART guidelines were published, in the fourth quarter of 2010, a total of 5352 were initiated on ART. In the next quarter – Jan-Mar 2011 – almost 6000 patients were initiated on ART. The average number of patients newly started on ART for these two quarters – 5,676 – was greater than the average of the previous period by 60 %. A TIPC report concerning the safety of NVP, showed that HIV infected patients were initiated on ART when their baseline CD4 cell count was >200cells/mm^3^ [“Personal Communications” – Dr. Assegid Mengistu]. Also, the analysis of NVP-related reaction reports showed that the majority of those who had the baseline CD4 cell counts recorded on the report (*n* = 55), were females (*n* = 48). Of these, 60 % had baseline CD4 cell counts >250 cells/mm^3^. It is likely that the increase in the number of patients initiated on ART was a result of initiating NVP-containing ART in patients with a CD4 cell count >200cells/mm^3^.

The increase in NVP-to-PI substitutions that was observed from the third quarter of 2010 to the third quarter of 2011 started with the use of TDF/3TC/NVP (Fig. 4). Since the new guidelines had lifted the prohibition of the use of NVP in females with baseline CD4 counts >250cells/mm^3^, plus the fact that female patients were indeed given NVP-containing first line ART with CD4 counts >250cells/mm^3^, we believed that the increase in NVP-to-PI substitutions was associated with the high baseline CD4 cell count in females. However, the number of patients for whom NVP was replaced with a PI was low, despite the statistical significance. We supposed that the few NVP-to-PI substitutions were because only a handful of female patients had a baseline CD4 cell count >250cells/mm^3^ at the start of TDF/3TC/NVP. This supposition borrowed evidence from the prospective study that TIPC carried out to assess the safety of first line ART, which showed that indeed many patients were initiated on ART with a CD4 cell count <250cells/mm^3^ [“Personal Communication” – Dr. Assegid Mengistu]. The reduction in the number of patients newly started on ART, coupled with the decline in the number of NVP-to-PI substitutions, may be due to reversion of HCW to the previous ART guidelines in regards to the safety of NVP.

Our findings were limited by the following reasons. First: We eliminated all records of patients who were changed from NVP to, and remained on EFV. This impacts on the results, because it is possible that NVP was substituted with EFV following ARs of any severity level – grades 1 to 4 – without recurrence or escalation of the reaction. For a sustained, unrelenting grade I reaction, or grade II reaction, the substitution of NVP with EFV is appropriate [20]. However, for grade III or IV reactions, the change to EFV is not recommended [21]. On the other hand, it was worth eliminating these since a change from NVP to EFV may have been implemented because of the management of tuberculosis, but this was not verifiable, by virtue of the data source that was employed. Second: we eliminated records of patients who were changed from NVP to LPV/r through EFV. We eliminated these, because we did not know whether the change to LPV/r was due to a reaction associated with EFV or it was the worsening of NVP’s reaction. Therefore, the elimination of records may have downplayed the proportion of NVP related reactions. The increase in the number of NVP-to-PI substitutions could be used to explain the increase in NVP-related skin and liver AR reports that were shown in the analysis of NVP-related AR reports [18]. Another limitation to this study was the lack of baseline CD4 count data. This data would have solidified our argument. Nevertheless, the argument stood the test as the new ART guidelines reverted back to the former safety measures: to avoid NVP in females with baseline CD4 counts above 250cells/mm^3^.

## Conclusion

The incidence of NVP-related reactions of grade III and IV increased with the implementation of Namibia’s 2010 ART Guidelines, which recommended the use of NVP in females with baseline (pre-ART) CD4 cell counts >250 cells/mm^3^. Therefore, the NVP safety signal was indeed a true signal. We proposed that initiating NVP-containing ART in women with high baseline CD4 counts, that is >250cells/mm^3^, was a key factor in the incidence of NVP-related grade III and IV reactions. The decision to halt the initiation of NVP-containing ART in all pregnant females in Namibia with high baseline CD4 cell counts was a significant intervention made by the MoHSS through the use of pharmacovigilance information from TIPC. We published an article on this NVP’s safety concern, in which we recommended a review on the use of NVP in Namibia. Namibia’s newest ART guidelines – 2014 guidelines – recommend the avoidance of NVP-containing ART in females and males with a baseline CD4 count >250 and 400 cells/mm^3^, respectively. We cannot claim that our findings were the sole reason for the change in guidelines, but we can say that they were contributory. National pharmacovigilance activities can be protective to patients, thus there is need for governments to strengthen local pharmacovigilance practice.

## Appendix 1

**Table 5:** Number of adverse reactions related to antiretrovirals, nevirapine, and other medicines in 2011 and in the Previous Years (2008 - 2010)

Adverse Reaction Reports	2011	2008–2010 (previous years)	RR
	*n* = 481	*n* = 593	
Adverse reaction related to antiretroviral medicines	389 (80.9 %)	482 (81.3 %)	1
Total NVP reactions (related to skin and liver)			
Adverse reactions attributed to NVP	208 (43.2 %)	99 (16.7 %)	2.6
NVP skin reactions			
All skin reactions	150 (31.2 %)	70 (11.8 %)	2.6
Severe	109 (22.7 %)	33 (5.6 %)	4.1
Mild/moderate	41 (8.5 %)	37 (6.2 %)	1.4
NVP-liver reactions			
All liver reactions	58 (12.1 %)	29 (4.9 %)	2.5
Severe	41 (8.5 %)	13 (2.2 %)	3.9
Mild/moderate	13 (2.7 %)	14 (2.4 %)	1.1
Unknown grade	4 (0.83 %)	2 (0.34 %)	-
Other antiretroviral medicine reactions			
Adverse reactions attributed to other medicines	273 (56.8 %)	494 (83.3 %)	0.7

The severe skin reaction reports in 2011 included: Stevens-Johnson Syndrome (*n* = 54), Toxic Epidermal Necrolysis (*n* = 3), Erythema Multiforme Major (*n* = 1), Skin reactions with conjunctivitis and oral lesions (51). In the Previous Years the severe reactions included the same reactions but much fewer reports. Note: The adverse reaction reports that never provided any form of description of the type of reaction – e.g., skin rash, were regarded as mild or moderate. The severe liver reaction reports in 2011 included: High alanine aminotransferase i.e., above 5× the upper limit of normal (many were far above this), hyperbilirubinaemia, and some were reported as severe hepatotoxicity. Reports that never provided description of the liver reaction were considered to be mild or moderate

## Appendix 2

**Table 6:** Statistical analysis of the difference between proportions of NVP-related adverse reactions of skin and liver

			% (95 % CI)	
Adverse reaction type		2011	Previous years	*P*-value
ARV	Related reactions (all)	80.9 % (77.4 %–84.4 %)	81.3 % (78.1 %–84.5 %)	0.87
NVP-related	Skin and liver	43.2 % (38.7 %–47.7 %)	16.7 % (13.7 %–19.7 %)	<0.0001
	Skin (all)	31.2 % (27.0 %–35.5 %)	11.8 % (9.2 %–14.4 %)	<0.0001
	Skin: severe	22.7 % (18.9 %–26.5 %)	5.6 % (3.7 %%–7.5 %)	<0.0001
	Skin: mild/moderate	8.5 % (6.0 %–11.0 %)	6.2 % (4.2 %–8.2 %)	0.16
	Liver (all)	12.1 % (9.2 %–15.0 %)	4.9 % (3.1 %–6.7 %)	<0.0001
	Liver: severe	8.5 % (6.0 %–11.0 %)	2.2 % (1.0 %–3.4 %)	<0.0001
	Liver: mild/moderate	2.7 % (1.0 %–4.4 %)	2.4 % (1.2 %–3.6 %)	0.73
Other medicines	Skin and liver	5.0 % (3.0 %–7.0 %)	9.9 % (7.5 %–12.3 %)	0.002
NVP-related reactions for	Females	29.5 % (25.4 %–33.6 %)	12.5 % (9.8 %–15.2 %)	<0.0001
	Females: severe	21.6 % (17.9 %–25.3 %)	5.6 % (3.7 %–7.5 %)	<0.0001
	Females: skin-severe	17.3 % (13.9 %–20.7 %)	4.0 % (2.4 %–5.6 %)	<0.0001
	Females: liver-severe	4.3 % (2.5 %–6.1 %)	1.5 % (0.5 %–2.5 %)	0.0072
	Males	13.7 % (10.6 %–16.8 %)	3.9 % (2.3 %–5.5 %)	<0.0001
	Males: severe	9.6 % (6.9 %–12.3 %)	2.2 % (1.0 %–3.4 %)	<0.0001
	Males: skin-severe	5.4 % (3.4 %–7.4 %)	1.5 % (0.5 %–2.5 %)	0.0007
	Males: liver-severe	4.2 % (2.4 %–6.0 %)	0.7 % (0.0 %–1.4 %)	0.0003

The results in the above table show that there was an increase in proportion of reports of severe reactions to NVP, on the skin and liver. The results also show that there was no increase in the mild and moderate reactions

## Appendix 3: Comparison of NVP-related reaction reports with reports of adverse effects for EFV and other Medicines

### Comparison of proportions of NVP- with EFV- related reactions

The Reaction Ratio (RR) for NVP was greater than that for EFV for both skin and liver associated reactions.

**Table 7:** RRs for NVP- and EFV- related adverse reaction reports

Category of reaction	NVP	EFV
Skin	2.6	1.9
Liver	2.5	0.4

### Comparison of proportions of NVP- and EFV- related reactions with other medicines

The Proportional Reporting Ratio (PRR) values for NVP were greater than those for EFV.

**Table 8:** PRR for NVP- and EFV- related adverse reaction reports

Category of reaction	PRR for NVP		PRR for EFV	
	2011	Previous years	2011	Previous years
Skin	3.8	1.5	0.06	0.06
Liver	9.7	2.2	0.03	0.17

## Abbreviations

3TC: lamivudine
AMR: adverse medicine reaction
AR: adverse reaction
ART: antiretroviral therapy
ARV: antiretroviral
CI: confidence interval
DHHS: Department of Health and Human Services
NDB: National antiretroviral Dispensing Database
FTC: emtricitabine
HAART: highly active antiretroviral therapy
HCW: health care workers
HIV: human immunodeficiency virus
MOHSS: Ministry of Health and Social Services
NRTI: nucleoside reverse transcriptase inhibitors
NVP: nevirapine
OR: Odds Ratio
PI: protease inhibitor
SD: standard deviation
TDF: tenofovir
TIPC: Therapeutics Information and Pharmacovigilance Centre
WHO: World Health Organization
